# Viruses as Modulators of Mitochondrial Functions

**DOI:** 10.1155/2013/738794

**Published:** 2013-10-24

**Authors:** Sanjeev K. Anand, Suresh K. Tikoo

**Affiliations:** ^1^Vaccine & Infection Disease Organization-International Vaccine Center (VIDO-InterVac), University of Saskatchewan, 120 Veterinary Road, Saskatoon, SK, Canada S7E 5E3; ^2^Veterinary Microbiology, University of Saskatchewan, 120 Veterinary Road, Saskatoon, SK, Canada S7E 5E3; ^3^School of Public Health, University of Saskatchewan, 120 Veterinary Road, Saskatoon, SK, Canada S7E 5E3

## Abstract

Mitochondria are multifunctional organelles with diverse roles including energy production and distribution, apoptosis, eliciting host immune response, and causing diseases and aging. Mitochondria-mediated immune responses might be an evolutionary adaptation by which mitochondria might have prevented the entry of invading microorganisms thus establishing them as an integral part of the cell. This makes them a target for all the invading pathogens including viruses. Viruses either induce or inhibit various mitochondrial processes in a highly specific manner so that they can replicate and produce progeny. Some viruses encode the Bcl2 homologues to counter the proapoptotic functions of the cellular and mitochondrial proteins. Others modulate the permeability transition pore and either prevent or induce the release of the apoptotic proteins from the mitochondria. Viruses like Herpes simplex virus 1 deplete the host mitochondrial DNA and some, like human immunodeficiency virus, hijack the host mitochondrial proteins to function fully inside the host cell. All these processes involve the participation of cellular proteins, mitochondrial proteins, and virus specific proteins. This review will summarize the strategies employed by viruses to utilize cellular mitochondria for successful multiplication and production of progeny virus.

## 1. Introduction 

### 1.1. Mitochondria

Mitochondria are cellular organelles found in the cytoplasm of almost all eukaryotic cells. One of their important functions is to produce and provide energy to the cell in the form of ATP, which help in proper maintenance of the cellular processes, thus making them indispensable for the cell. Besides acting as a powerhouse for the cell, they act as a common platform for the execution of a variety of cellular functions in normal or microorganism infected cells. Mitochondria have been implicated in aging [1, 2], apoptosis [3–7], the regulation of cell metabolism [4, 8], cell-cycle control [9–11], development of the cell [12–14], antiviral responses [15], signal transduction [16], and diseases [17–20].

Although all mitochondria have the same architecture, they vary greatly in shape and size. The mitochondria are composed of outer mitochondrial membrane, inner mitochondrial membrane, intermembrane space (space between outer and inner membrane), and matrix (space within inner mitochondrial membrane). The outer membrane is a smooth phospholipid bilayer, with different types of proteins imbedded in it [21]. The most important of them are the porins, which freely allow the transport (export and import) of the molecules (proteins, ions, nutrients, and ATP) less than 10 kDa across the membranes. The outer membrane surrounds the inner membrane creating an intermembrane space that contains molecules such as Cyt-C, SMAC/Diablo, and endonuclease G. It also acts as a buffer zone between the outer membrane and the inner membrane of mitochondria. The inner membrane is highly convoluted into structures called cristae, which increases the surface area of the membrane and are the seats of respiratory complexes. The inner membrane of mitochondria allows the free transport of oxygen and carbon dioxide. The movement of water through membranes is suggested to be controlled by aquaporins channel protein [22, 23] though a report suggested otherwise [24]. The matrix contains enzymes for the aerobic respiration, dissolved oxygen, water, carbon dioxide, and the recyclable intermediates that serve as energy shuttles and perform other functions.

Mitochondria contain a single 16 kb circular DNA genome, which codes for 13 proteins (mostly subunits of respiratory chains I, II, IV, and V), 22 mitochondrial tRNAs and 2 rRNAs [25, 26]. The mitochondrial genome is not enveloped (like nuclear envelop), contains few introns, and does not follow universal genetic code [27]. Although the majority of the mitochondrial proteins are encoded by nuclear DNA and imported into the mitochondria (reviewed by [21, 28–31]), mitochondria synthesize few proteins that are essential for their respiratory function [1, 27]. 

Proteins destined to mitochondria have either internally localized [28] or amino terminal localized [21] presequences known as mitochondria/matrix localization signals (MLS), which can be 10–80 amino acid long with predominantly positively charged amino acids. The combination of these presequences with adjacent regions determines the localization of a protein in respective mitochondrial compartments. The outer mitochondrial membrane contains two major translocators, namely, (a) the translocase of outer membrane (TOM) 40, which functions as an entry gate for most mitochondrial proteins with MLS and (b) sorting and assembly machinery (SAM) or translocase of *β*-barrel (TOB) protein, which is a specialized insertion machinery for beta-barrel membrane proteins [32]. Once proteins pass through the outer membrane, they are recruited by presequence translocase-associated motor (PAM) to the translocase of the inner mitochondrial membrane (TIM) 23 complexes, which mediates the import of proteins to the matrix. Finally, the presequences are cleaved in matrix and proteins are modified to their tertiary structure, and rendered functional [30].

### 1.2. Viruses

**Figure 1:**
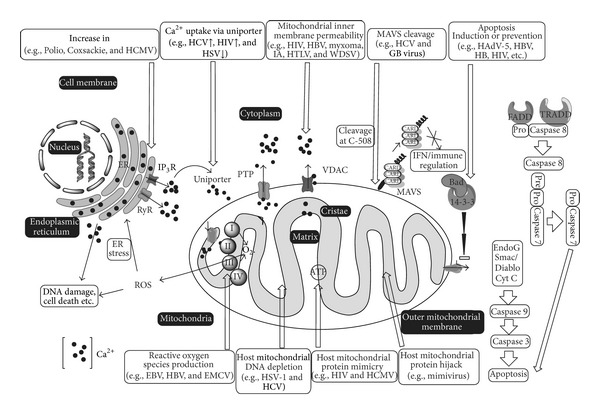
Schematic diagram of cell showing mitochondria, nucleus endoplasmic reticulum (ER) and cell membrane. iCa^2+^: intracellular calcium, FADD: Fas-associated protein with death domain, TRADD: tumor necrosis factor receptor type 1-associated death domain protein, PTP: permeability transition pore, VDAC: voltage-dependent anion channel, IP_3_R: inositol 1,4,5-trisphosphate receptor, RyR: ryanodine receptor, MAVS: mitochondrial antiviral signaling, I, II, III, and IV are complex I to IV of electron transport chain. O_2_^−^: Superoxide radical, Bad, Bcl-2-associated death promoter, ROS: reactive oxygen species, IFN: interferon, HCMV: human cytomegalovirus, HIV: human immunodeficiency virus, HSV: herpes simplex virus, HBV: hepatitis B virus, HTLV: human T-lymphotropic virus, IA: influenza A virus, WDSV: Walleye dermal sarcoma virus, HCV: hepatitis C virus, HAdV: human adenovirus-5, EBV: Epstein-Barr virus, and EMCV: encephalomyocarditis virus.

Viruses are acellular obligate intracellular microorganisms that infect the living cells/organisms and are the only exception to cell theory proposed by Schleiden and Schwann in 1838/1839 [33]. The viruses have an outer protein capsid and a nucleic acid core. Usually, the viral nucleic acids can be either DNA (double or single stranded) or RNA (+ or − sense single stranded or double stranded RNA). Some of the viruses are covered with an envelope embedded with glycoproteins. The viruses have long been associated with the living organisms, and it was in the later part of the century that their relationship with various cellular organelles was studied in detail. In order to survive and replicate in the cell, viruses need to take control of the various cellular organelles involved in defense and immune processes. They also require energy to replicate and escape from the cell. Once inside the host cell, they modulate various cellular signal pathways and organelles, including mitochondria, and use them for their own survival and replication. This review summarizes the functions of mitochondria and how viruses modulate them (Figure 1).

## 2. Viruses Regulate Ca^2+^ Homeostasis in Host Cells

### 2.1. Ca^2+^ Homeostasis

Ca^2+^ is one of the most abundant and versatile elements in the cell and acts as a second messenger to regulate many cellular processes [34]. Earlier, outer membrane of mitochondria was thought to be permeable to Ca^2+^, but recent studies suggest that the outer membrane contains voltage-dependent anion channels (VDAC) having Ca^2+^ binding domains, which regulate the entry of Ca^2+^ into the mitochondrial intermembrane space [35–37]. The influx of Ca^2+^ through the inner membrane is regulated by the mitochondrial Ca^2+^ uniporter (MCU), which is a highly selective Ca^2+^ channel that regulates the Ca^2+^ uptake based on mitochondrial membrane potential (MMP). The net movement of charge due to Ca^2+^ uptake is directly proportional to the decrease of MMP [38]. A second mechanism that helps in Ca^2+^ movement across the mitochondria membrane is called “rapid mode” uptake mechanism (RaM) [39]. In this process, Ca^2+^ transports across the mitochondrial membrane by exchange with Na^+^, which in turn depends upon its exchange with H^+^ ion and thus MMP. This ion exchange across the mitochondrial membrane decreases MMP and is dependent on electron transport chain (ETC) for its maintenance. A third mechanism involves IP_3_R, a Ca^2+^ channel in endoplasmic reticulum. IP_3_R is connected to mitochondrial VDAC through a glucose regulating protein 75 (GRP75). This junction regulates/facilitates Ca^2+^ exchange from IP_3_R to VDAC [40].

Ca^2+^ efflux mechanism is regulated by the permeability transition pore (PTP). The PTP is assembled in the mitochondrial inner and outer membranes [41, 42], with Ca^2+^ binding sites on the matrix side of the inner membrane. The PTP regulates the mitochondrial Ca^2+^ release by a highly regulated “Flickering” mechanism that controls the opening and closing of the pore [43]. RaM works in sync with ryanodine receptor (RyR) isoform 1, which is another very important calcium release channel [44]. Both RyR and RaM regulate the phenomenon of excitation-metabolism coupling in which cytosolic Ca^2+^ induced contraction is matched by mitochondrial Ca^2+^ stimulation of ox-phos [45]. However, mitochondrial Ca^2+^ overload can result in prolonged opening of the pore leading to pathology [46]. Although Ca^2+^ is involved in the activation of many cellular processes including stimulation of the ATP synthase [47, 48], allosteric activation of Krebs cycle enzymes [49, 50], and the adenine nucleotide translocase (ANT) [51], the primary role of mitochondrial Ca^2+^ is in the stimulation of ox-phos [52–54]. Thus, the elevated mitochondrial Ca^2+^ results in up regulation of the entire ox-phos machinery, which then results in faster respiratory chain activity and higher ATP output, which can then meet the cellular ATP demand. Ca^2+^ also upregulates other mitochondrial functions including activation of N-acetylglutamine synthetase to generate N-acetylglutamine [55], potent allosteric activation of carbamoyl-phosphate synthetase, and the urea cycle [56]. Thus, any perturbation in mitochondrial or cytosolic Ca^2+^ homeostasis has profound implications for cell function. Moreover, mitochondrial Ca^2+^, particularly at high concentrations experienced in pathology appears to have several negative effects on mitochondrial functions [57].

### 2.2. Regulation by Viruses

A number of viruses alter the Ca^2+^regulatory activity of the cell for their survival. Herpes simplex type (HSV) 1 virus causes a gradual decline (65%) in mitochondrial Ca^2+^ uptake at 12 hrs lytic cycle [58], which helps in virus replication. Although mitochondrial Ca^2+^ uptake keeps fluctuating throughout the course of a measles virus infection of cells, the total amount of cellular Ca^2+^ remains the same [58] indicating the tight control that the virus exerts over the cellular processes during its life cycle. 

The core protein of hepatitis C virus (HCV) targets mitochondria and increases Ca^2+^ [59, 60]. The NS5A protein of HCV causes alterations in Ca^2+^ homeostasis [61–63]. Both of these proteins may be responsible for the pathogenesis of liver disorders associated with HCV infection. Even in the cells coinfected with HCV and human immunodeficiency virus (HIV), these viruses enhance the MCU activity causing cellular stress and apoptosis [59, 64]. The p7 protein of HCV forms porin-like structures [65] and causes Ca^2+^ influx to cytoplasm from storage organelles [66]. These HCV proteins disturb the Ca^2+^ homeostasis at different stages of the infection and thus help to enhance the survival of the cell. Interestingly, interaction of protein X of hepatitis B virus (HBV) with VDAC causes the release of Ca^2+^ from storage organelles mitochondria/endoplasmic reticulum (ER)/golgi into the cytoplasmic compartment, which appears to help virus replication [67, 68].

The *Nef *protein of HIV interacts with IP_3_R [69] and induces an increase in cytosolic Ca^2+^ through promotion on T cell receptor-independent activation of the NFAT pathway [70]. Activated NFAT, in turn, causes the low-amplitude intracellular Ca^2+^ oscillation, promoting the viral gene transcription and replication [71]. 

Ca^2+^ is an important factor for different stages of rotavirus lifecycle and for stability to rotavirus virion [72]. The NSP4 protein of rotavirus increases the cytosolic Ca^2+^ concentration by activation of phospholipase C (PLC) and the resultant ER Ca^2+^ depletion through IP_3_R [73, 74]. This alteration in Ca^2+^ homeostasis has been attributed to an increase in the permeability of cell membrane [75]. A decrease in cellular Ca^2+^ concentrations toward the end of the life cycle has been reported to enable rotavirus release from the cell [76].

The 2BC protein of poliovirus increases the intracellular Ca^2+^ concentrations in the cells 4 hrs. After infection, which is necessary for viral gene expression [77, 78]. Toward the end of the virus life cycle, the release of Ca^2+^ from the lumen of ER through IP_3_R and RyR channels causes accumulation of Ca^2+^ in mitochondria through uniporter and VDAC resulting in mitochondrial dysfunction and apoptosis [79]. On the contrary, the 2B protein of Coxsackie virus decreases the membrane permeability by decreasing Ca^2+^ concentrations in infected cells [80, 81] due to its porin-like activity that results in Ca^2+^ efflux from the organelles. Reduced protein trafficking and low Ca^2+^ concentration in golgi and ER favor the formation of viral replication complexes, downregulate host antiviral immune response, and inhibit apoptosis [82, 83]. 

Enteroviruses orchestrate the apoptotic process during their life cycle to enhance its entry, survival, and release. The perturbation in cytoplasmic Ca^2+^ homeostasis at 2–4 hrs. postinfection coincides with the inhibition of the apoptotic response that can be attributed to decrease in cytotoxic levels of Ca^2+^ in the cell and the mitochondria. This also provides the virus with optimum conditions for the replication and protein synthesis. Finally, a decrease in mitochondrial and other storage organelles (ER and golgi) Ca^2+^ levels causes an increase in cytosolic Ca^2+^ concentration, leading to the formation of vesicles and cell death, thus assisting in virus release [81, 84, 85].

The pUL37 × 1 protein of human cytomegalovirus (HCMV) localizes to mitochondria [86] and causes the trafficking of Ca^2+^ from the ER to mitochondria at 4–6 hrs. After infection [87]. Active Ca^2+^ uptake by mitochondrion induces the production of ATP and other Ca^2+^ dependent enzymes accelerating virus replication, and a decrease in Ca^2+^ levels in the ER has antiapoptotic effects [88]. 

The 6.7K protein encoded by E3 region of HAdV-2 localizes to ER and helps maintain ER Ca^2+^ homeostasis in transfected cells, thus inhibiting apoptosis [89].

## 3. Viruses Cause Oxidative Stress in Host Cells

### 3.1. Electron Transport Chain

The mitochondrial respiratory chain is the main and most significant source of reactive oxygen species (RO) in the cell. Superoxide (O_2_^−•^) is the primary ROS produced by mitochondria. In the normal state, there is little or no leakage of electrons between the complexes of the electron transport chain (ETC). However, during stress conditions, a small fraction of electrons leave complex III and reach complex IV [90]. This premature electron leakage to oxygen results in the formation of two types of superoxides, namely, O_2_^−^, in its anionic form, and HO_2_^−^ in its protonated form. 

Leakage of electrons takes place mainly from Q_O_ sites of complex III, which are situated immediately next to the intermembrane space resulting in the release of superoxides in either the matrix or the innermembrane space of the mitochondria [91–94]. About 25–75% of the total electron leak through Complex III could account for the net extramitochondrial superoxide release [95–97]. Thus, the main source of O_2_^−•^ in mitochondria is the ubisemiquinone radical intermediate (QH^•^) formed during the Q cycle at the Q_O_ site of complex III [98–100]. Complex I is also a source of ROS, but the mechanism of ROS generation is less clear. Recent reports suggest that glutathionylation [101] or PKA mediated phosphorylation [101–103] of complex I can elevate ROS generation. Backward flow of electron from complex I to complex II can also result in the production of ROS [99].

A variety of cellular defense mechanisms maintain the steady state concentration of these oxidants at nontoxic levels. This delicate balance between ROS generation and metabolism may be disrupted by various xenobiotics including viral proteins. The main reason for generation of ROS in virus-infected cells is to limit the virus multiplication. However, ROS also acts as a signal for various cellular pathways, and the virus utilizes the chaos generated inside the cell for its replication. 

### 3.2. Viruses Induce Reactive Oxygen Species

A number of viruses cause oxidative stress to the host cells, which directly or indirectly helps them to survive. Human-Adenovirus- (HAdV-) 5 has been reported to induce the rupture of endosomal membrane upon infection resulting in the release of lysosomal cathepsins, which prompt the production of ROS. Cathepsins also induce the disruption of mitochondrial membrane leading to the release of ROS from mitochondria thus causing the oxidative stress [104]. 

The core protein of HCV causes oxidative stress in the cell and alters apoptotic pathways [64, 105–107]. The E1, E2, NS3, and core protein of HCV are potent ROS inducers and can cause host DNA damage independently [107, 108] or mediated by nitric oxide (NO) thus aiding in virus replication.

The ROS is generated during HIV infection [64, 109–111]. H_2_O_2_, an ROS generated during HIV infection strongly induces HIV long terminal repeat (LTR) via NF-kappa B activation. Impaired LTR activity ablates the LTR activation in response to ROS thus aiding in virus replication [112]. HIV also causes extensive cellular damage due to increased ROS production and decreased cytosolic antioxidant production [113]. Coinfection of HIV and HCV causes the hepatic fibrosis, the progression of which is regulated through the generation of ROS in an NF-*κ*B dependent manner [113].

Epstein-Barr virus (EBV) causes increased oxidative stress in the host cells within 48 hrs. During the lytic cycle indicating the role of ROS in virus release [114]. Oxidative stress activates the EBV early gene BZLF-1, which causes the reactivation of EBV lytic cycle [114]. This has been proposed to play an important role in the pathogenesis of EBV-associated diseases including malignant transformations [115, 116]. 

Interestingly, HBV causes both an increase and a decrease in oxidative stress to enhance its survival in the host cells [117, 118]. HBV induces strong activation of Nrf2/ARE-regulated genes *in vitro* and *in vivo* through the activation of c-Raf and MEK by HBV protein X thus protecting the cells from HBV induced oxidative stress and promoting establishment of the infection [119]. The protein X of HBV also induces the ROS mediated upregulation of Forkhead box class O4 (Foxo4), enhancing resistance to oxidative stress-induced cell death [120]. However, reports also suggest that upon exposure to oxidative stress, HBV protein X accelerates the loss of Mcl-1 protein via caspase-3 cascade thus inducing pro apoptotic effects [118]. Coinfection of HCV also causes the genotoxic effects in peripheral blood lymphocytes due to increased oxidative damage and decreased MMP [121]. It is possible that contradictory functions of protein X of HBV cold occur at different stages of virus replication.

Encephalomyocarditis virus (EMCV) causes oxidative stress in the cells during infection damaging the neurons, which is an important process in the pathogenesis of EMCV infection [122]. 

## 4. Viruses Regulate Mitochondrial Membrane Potential in Host Cells

### 4.1. Mitochondrial Membrane Potential

Membrane potential (MP) is the difference in voltage or electrical potential between the interior and the exterior of a membrane. The membrane potential is generated either by electrical force (mutual attraction or repulsion between both positive or negative) and/or by diffusion of particles from high to low concentrations. The mitochondrial membrane potential (MMP) is an MP (≅ 180 mV) across the inner membrane of mitochondria, which provides energy for the synthesis of ATP. Movement of protons from complex I to V of electron transport chain (ETC) located in the inner mitochondrial membrane creates an electric potential across the inner membrane, which is important for proper maintenance of ETC and ATP production. Reported MMP values for mitochondria (*in vivo*) differ from species to species and from one organ to another depending upon the mitochondria function, protein composition, and the amount of oxidative phosphorylation activity required in that part of the body [43].

The voltage dependent anionic channels (VDACs) also known as mitochondrial porins form channels in the outer mitochondrial membranes and act as primary pathway for the movement of metabolites across the outer membrane [37, 96, 123–125]. In addition, a number of factors including oxidative stress, calcium overload, and ATP depletion induce the formation of nonspecific mitochondrial permeability transition pores (MPTP) in the inner mitochondrial membrane, which is also responsible for the maintenance of MMP [36, 37, 126]. The outer membrane VDACs, inner membrane adenine nucleotide translocase (ANT) [127], and cyclophilin D (CyP-D) in matrix are the structural elements of the mitochondrial permeability transition pore (MPTP).

When open, MPTP increases the permeability of the inner mitochondrial membrane to ions and solutes up to 1.5 kDa, which causes dissipation of the MMP and diffusion of solutes down their concentration gradients, by a process known as the permeability transition [128, 129]. The MPTP opening is followed by osmotic water flux, passive swelling, outer membrane rupture, and release of proapoptotic factors leading to the cell death [42, 130]. Because of the consequent depletion of ATP and Ca^2+^ deregulation, opening of the MPTP had been proposed to be a key element in determining the fate of the cell before a role for mitochondria in apoptosis was proposed [129].

The MMP can be altered by a variety of stimuli including sudden burst of ROS [43, 107], Ca^2+^ overload in the mitochondria or the cell [48, 57, 131], and/or by proteins of invading viruses [109, 132, 133]. In general, an increase or decrease in MMP is related to the induction or prevention of apoptosis, respectively. Prevention of apoptosis during early stages of virus infection is a usual strategy employed by viruses to prevent host immune response and promote their replication. On the contrary, induction of apoptosis during later stages of virus infection is a strategy used by viruses to release the progeny virions for dissemination to the surrounding cells. 

### 4.2. Regulation by Viruses

Many viral proteins alter mitochondrial ion permeability and/or membrane potential for their survival in the cell. The p7, a hydrophobic integral membrane [134] viroprotein [135] of HCV, localizes to mitochondria [66] and controls membrane permeability to cations [66, 136] promoting cell survival for virus replication [135]. 

The R (Vpr) protein of HIV, a small accessory protein, localizes to the mitochondria, interacts with ANT, modulates MPTP, and induces loss of MMP promoting release of Cyto C [137] leading to cell death [138, 139]. The Tat protein of HIV also modulates MPTP leading to the accumulation of Tat in mitochondria and induction of loss of MMP resulting in caspase dependent apoptosis [140]. 

The M11L protein of myxoma poxvirus localizes to the mitochondria, interacts with the mitochondrial peripheral benzodiazepine receptor (PBR), and regulates MPTP [141] inhibiting MMP loss [142] and thus inhibiting induction of apoptosis during viral infection [143]. The FIL protein of vaccinia virus downregulates proapoptotic Bcl-2 family protein Bak and, inhibits the loss of the MMP and the release of Cyt-C [144, 145]. The crmA/Spi-2 protein of vaccinia virus, a caspase 8 inhibitor, modulates MPTP thus preventing apoptosis [146].

The PB1-F2 protein of influenza A viruses localizes to the mitochondria [147–150] and interacts with VDAC1 and ANT3 [151] resulting in decreased MMP, which induces the release of proapoptotic proteins causing cell death. Recent evidence shows that PB1-F2 is also able to form nonselective protein channel pores resulting in the alteration of mitochondrial morphology, dissipation of MMP, and cell death [150]. The M2 protein of influenza virus, a viroprotein, causes the alteration of mitochondrial morphology, dissipation of MMP, and cell death (reviewed by [135]).

The p13II, an accessory protein encoded by x-II ORF of human T-lymphotropic virus (HTLV), a new member of the viroprotein family [152], localizes to the mitochondria of infected cells and increases the MMP leading to apoptosis [153] and mitochondrial swelling [153–155]. 

The Orf C protein of Walleye dermal sarcoma virus (WDSV) localizes to the mitochondria [156] and induces perinuclear clustering of mitochondria and loss of MMP [156] leading to the release of proapoptotic factors thus causing apoptosis. 

The 2B protein of Coxsackie virus decreases MMP by decreasing the Ca^2+^ concentrations in infected cells [80, 81]. 

## 5. Viruses Regulate Apoptosis

### 5.1. Apoptosis

During the coevolution of viruses with their hosts, viruses have developed several strategies to manipulate the host cell machinery for their survival, replication, and release from the cell. Viruses target the cellular apoptotic machinery at critical stages of viral replication to meet their ends [157, 158]. Depending upon the need, a virus may inhibit [159] or induce [160] apoptosis for the obvious purpose of replication and spread, respectively [158, 159]. Interference in mitochondrial function can cause either cell death due to deregulation of the Ca^2+^ signaling pathways and ATP depletion or apoptosis due to regulation of Bcl-2 family proteins. Apoptosis is a programmed cell death [161] characterized by membrane blebbing, condensation of the nucleus and cytoplasm, and endonucleosomal DNA cleavage. The process starts as soon as the cell senses physiological or stress stimuli, which disturbs the homeostasis of the cell [162, 163]. Apoptotic cell death can be considered as an innate response to limit the growth of microorganisms including viruses attacking the cell. 

Two major pathways, namely, the extrinsic and the intrinsic are involved in triggering apoptosis [163, 164]. The extrinsic pathway is mediated by signaling through death receptors like tumor necrosis factor or Fas ligand receptor causing the assembly of death inducing signaling complex (DISC) with the recruitment of proteins like caspases leading to the mitochondrial membrane permeabilization. In the intrinsic pathway, the signals act directly on the mitochondria leading to mitochondrial membrane permeabilization before caspases are activated causing the release of Cyt-C [165, 166], which recruits APAF1 [167, 168] resulting in direct activation of caspase 9 [35, 169]. Both the extrinsic and the intrinsic processes congregate at the activation of downstream effector caspases, (i.e., caspase-3) [170] which is responsible for inducing the morphological changes observed in an apoptotic cell. In addition to Cyt-C, Smac/DIABLO as well as caspase independent death effectors inducing factor (AIF) and endonuclease G [171–173] acts as an activator of the caspase. 

The B cell lymphoma- (Bcl-) 2 family of proteins tightly regulate the apoptotic events involving the mitochondria [174, 175]. More than 20 mammalian Bcl-2 family proteins have been described to date [176, 177]. They have been classified by the presence of Bcl-2 homology (BH) domains arranged in the order BH4-BH3-BH2-BH1 and the C-terminal hydrophobic transmembrane (TM) domain, which anchors them to the outer mitochondrial membrane [178]. The highly conserved BH1 and BH2 domains are responsible for antiapoptotic activity and multimerization of Bcl-2 family proteins. The BH3 domain is mainly responsible for proapoptotic activity and the less conserved BH4 domain is required for the antiapoptotic activities of Bcl-2 and Bcl-X_L_ proteins [174, 178]. Most of the antiapoptotic proteins are multidomain proteins, which contain all four BH domains (BH1 to BH4) and a TM domain. In contrast, proapoptotic proteins are either multidomain proteins, which contain three BH domains (BH1 to BH3) or single domain proteins, which contain one domain (BH3) [158]. The Bcl-2 proteins regulate the MMP depending upon whether they belong to the pro- or antiapoptotic branch of the family, respectively. The MMP marks the dead end of apoptosis beyond which cells are destined to die [125, 166, 179–183]. 

### 5.2. Regulation by Viruses

Viruses encode homologs of Bcl-2 (vBcl-2) proteins, which can induce (pro-apototic) or prevent (antiapoptotic) apoptosis thus helping viruses to complete their life cycle in the host cells [117, 163, 175]. While the vBcl-2s and the cellular Bcl-2s share limited sequence homology, their secondary structures are predicted to be quite similar [158, 174, 184]. During primary infection, interplay between vBcl-2 and other proteins enhances the lifespan of the host cells resulting in efficient production of viral progeny and ultimately spread of infection to the new cells. It also favors viral persistence in the cells by enabling the latently infecting viruses to make the transition to productive infection. The pathways and strategies used by viruses to induce/inhibit apoptosis have been reviewed earlier [185]. 

Many viruses encode for the homologs of antiapoptotic Bcl-2 proteins, which preferentially localize to the mitochondria and may interact with the other proapoptotic Bax homologues. The E1B19K encoded by human-adenovirus- (HAdV-) 5 contains BH1 and BH3-like domains and blocks TNF-alpha-mediated death signaling by inhibiting a form of Bax that interrupts the caspase activation downstream of caspase-8 and upstream of caspase-9 [186, 187]. Like HAdV-5 E1B19K [186], some viruses encode Bcl-2 homologues lacking BH4 domain, which are thought to act by inhibiting proapoptotic members of Bcl-2 family proteins. The FPV309 protein encoded by fowl pox virus contains highly conserved BH1 and BH2-like domains, and a cryptic BH3 domain, interacts with Bax protein and inhibits apoptosis [188]. The A179L protein encoded by African swine fever virus (ASFV) contains BH1 and BH2 domains and, interacts with Bax-Bak proteins and inhibits apoptosis [189, 190]. The Bcl-2 homolog (vBcl-2) encoded by Herpesvirus saimiri (HVS) contains BH3 and BH4-like domains and interacts with Bax, thus stabilizing mitochondria against a variety of apoptotic stimuli preventing the cell death [191]. The E4 ORF encoded by equine Herpesvirus-3 contains BH1 and BH2 domains [192], which may interact with Bax and be essential for antiapoptotic activity [193].

Viruses also encode homologs of proapoptotic Bcl-2 proteins. The HBV encodes protein X, a vBcl-2 protein containing BH3, which localizes to the mitochondria and interacts with VDACs inducing the loss of the MMP leading to apoptosis [117, 121, 194, 195] or interacts with Hsp60 and induces apoptosis [196]. In contrast, another study revealed the protective effects of HB-X in response to proapoptotic stimuli (Fas, TNF, and serum withdrawal) but not from chemical apoptotic stimuli [197]. The protein X of HBV is known to stimulate NF*κ*B [198, 199], SAPK [200, 201], and PI3K/PKB [202] to prevent apoptosis. It is possible that the diverse functions of HBV protein X occur at different times of virus replication cycle in the infected cells. The BALF1 protein encoded by EBV contains BH1 and BH4 domains [203], which interacts with the Bax-Bak proteins [192] and inhibits the antiapoptotic activity of the EBV BHRF1 and the Kaposi Sarcoma virus (KSV) Bcl-2 protein, both of which contain BH1 and BH2 domains [204] and interact with BH3 only proteins [205]. 

The effects of viral Bcl-2 homologues are thus apparently centered around mitochondria and include prevention or induction of MMP loss. The induction of MMP loss leads to the release of Cyto C and other proapoptotic signals into the cytosol and activation of downstream caspases leading to the cell death and dissemination of viruses to neighbouring cells for further infection.

Viruses encode pro/anti apoptotic proteins, which show no homology to Bcl-2 proteins [158]. The E6 protein of human papilloma virus (HPV) downregulates Bax signal upstream of mitochondria [206, 207] and prevents the release of Cyto C, AIF, and Omi, thus preventing apoptosis [208]. This E6 activity towards another Bcl2 family proapoptotic protein Bak is a key factor promoting the survival of HPV-infected cells, which in turn facilitates the completion of viral life cycle [207]. Enterovirus (EV) 71 induces conformational changes in Bax and increases its expression in cells following infection and induces the activation of caspases 3, 8, and PARP causing caspase dependent apoptosis [209]. On the contrary, Rubella viral capsid binds to Bax, forms oligoheteromers, and prevents the formation of pores on mitochondrial membrane thus preventing Bax induced apoptosis [210].

Viruses also encode proteins, which act as viral mitochondrial inhibitors of apoptosis (vMIA) thus protecting the cells. A splice variant of UL37 of HCMV acts as vMIA and protects the cells from apoptosis [211] thereby helping viruses to complete their replication cycle. It localizes to mitochondria and interacts with ANT [211] and Bax [212, 213]. HCMV vMIA has an N-terminal mitochondrial localization domain and a C-terminal antiapoptotic domain [211], which recruits Bax to mitochondria and prevents loss of MMP. It protects the cells against CD95 ligation [211] and oxidative stress-induced cell death [214, 215] and prevents mitochondrial fusion [216] thus promoting cell survival. 

vMIA does not inhibit the apoptotic events upstream of mitochondria but can influence events like preservation of ATP generation, inhibition of Cyto C release, and caspase 9 activation, following induction of apoptosis. However, the exact mechanisms of the events around vMIA still remain a question.

## 6. Viruses Modulate Mitochondrial Antiviral Immunity

### 6.1. Mitochondrial Antiviral Immunity

 Cells respond to virus attack by activating a variety of signal transduction pathways leading to the production of interferons [217], which limit or eliminate the invading virus. The presence of viruses inside the cell is first sensed by pattern recognition receptors (PRRs) that recognize the pathogen associated molecular patterns (PAMPs). PRRs include toll-like receptors (TLRs), nucleotide oligomerization domain (NOD) like receptors (NLRs), and retinoic acid-inducible gene I (RIG-I) like receptors (RLRs). Mitochondria have been associated with RLRs, which include retinoic acid-inducible gene I (RIG-I) [218] and melanoma differentiation-associated gene 5 (Mda-5) [219]. Both are cytoplasm-located RNA helicases that recognize dsRNA. The N-terminus of RIG-1 has caspase activation and recruitment domains (CARDs) whereas C-terminus has RNA helicase activity [218], which recognizes and binds to uncapped and unmodified RNA generated by viral polymerases in ATPase dependent manner. This causes conformational changes and exposes its CARD domains to bind and activate downstream effectors leading to the formation of enhanceosome [220] triggering NF*κ*B production. RLRs have recently been reviewed in detail [221–223].

A CARD domain containing protein named mitochondrial antiviral signaling (MAVS) [15, 224], virus-induced signaling adaptor (VISA) [225], IFN-*β* promoter stimulator 1 (IPS-1) [226], or CARD adaptor inducing IFN-*β* (CARDIF) protein [227] acts downstream of the RIG-I. Besides the presence of N-terminal CARD domain, MAVS contains a proline-rich region and a C-terminal hydrophobic transmembrane (TM) region, which targets the protein to the mitochondrial outer membrane and is critical for its activity [15]. The TM region of the MAVS resembles the TM domains of many C-terminal tail-anchored proteins on the outer membrane of the mitochondria including Bcl-2 and Bcl-xL [15]. Recent reports indicate that MAVS has an important role in inducing the antiviral defenses in the cell. Overexpression of MAVS leads to the activation of NF*κ*B and IRF-3, leading to the induction of type I interferon response, which is abrogated in the absence of MAVS [15] thus indicating the specific role of MAVS in inducing antiviral response. MAVS has also been shown to prevent apoptosis by its interaction with VDAC [228] and preventing the opening of MPTP. 

### 6.2. Regulation by Viruses

Some viruses induce cleavage of MAVs from outer membranes of mitochondria [227, 229] thus greatly reducing their ability to induce interferon response. HCV persists in the host by lowering the host cell immune response including inhibiting the production of IFN-*β* by RIG-I pathway [230–232]. The NS3/4A protein of HCV colocalizes with mitochondrial MAVS [227, 229] leading to the cleavage of MAVS at amino acid 508. Since free form of the MAVS is not functional, the dislodging of MAV from the mitochondria inactivates MAVS [227] thus helping in paralyzing the host defense against HCV. Interestingly, another member of family *Flaviviridae* GB virus B shares 28% amino acid homology with HCV over the lengths of their open-reading frames [233]. The NS3/4A protein of GB virus also cleaves MAVS in a manner similar to HCV, thus effectively compromising the host immune response by preventing the production of interferons [234]. Other viruses like influenza A translocate RIG-I/MAVS components to the mitochondria of infected human primary macrophages and regulate the antiviral/apoptotic signals increasing the viral survivability [235].

## 7. Viruses Hijack Host Mitochondrial Proteins

 Over the years, viruses have perfected different strategies to establish complex relationships with their host with the sole purpose of preserving their existence. One such strategy involves the hijacking of the host cell mitochondrial proteins. The p32, a mitochondria-associated cellular protein, is a member of a complex involved in the import of cytosolic proteins to the nucleus. Upon entry into the cell, adenovirus hijacks this protein and piggybacks it to transport its genome to the nucleus [236], thereby increasing its chances of survival and establishment in the host cell. During HIV-1 assembly, tRNA^Lys^ iso-acceptors are selectively incorporated into virions, and tRNA_3_^Lys^ binds to HIV genome and is used as the primer for reverse transcription [237]. In humans, a single gene produces both cytoplasmic and mitochondrial Lys tRNA synthetases (LysRSs) by alternative splicing [238]. The mitochondrial LysRS is produced as a preprotein, which is transported into the mitochondria. The premitochondrial or mitochondrial LysRS is specifically packaged into HIV [239] and acts as a primer to initiate the replication of HIV-I RNA genome, which then binds to a site complementary to the 3′-end 18 nucleotides of tRNA_3_^Lys^. It is proposed that HIV viral protein R (Vpr) alters the permeability of the mitochondria [138] leading to the release of premito- or mito-LysRS, which then interacts with Vpr [240] and gets packed into the progeny virions. 

Viperin, an interferon inducible protein, is induced in the cells in response to viral infection [241]. This protein has been shown to prevent the release of influenza virus particles from the cells by trapping them in lipid rafts inside the cells thereby preventing its dissemination [242]. During infection, HCMV induces IFN independent expression of viperin, which interacts with HCMV encoded vMIA protein resulting in relocation of viperin from ER to mitochondria. In mitochondria, viperin interacts with mitochondrial tri-functional protein and decreases ATP generation by disrupting oxidation of fatty acids, which results in disrupting actin cytoskeleton of the cells and enhancing the viral infectivity [243]. 

## 8. Viruses Alter Intracellular Distribution of Mitochondria

 Viruses alter the intracellular distribution of mitochondria either by concentrating the mitochondria near the viral factories to meet energy requirements during viral replication or by cordoning off the mitochondria within cytoplasm to prevent the release of mediators of apoptosis. The protein X of HBV causes microtubule mediated perinuclear clustering of the mitochondria by p38 mitogen-activated protein kinase (MAPK) mediated dynein activity [244]. HCV nonstructural protein 4A (NS4A) either alone or together with NS3, (in the form of the NS3/4A polyprotein) accumulates on mitochondria and changes their intracellular distribution [245]. HIV-1 infection causes clustering of the mitochondria in the infected cells [246]. Interestingly, ASFV causes the microtubule-mediated clustering of the mitochondria around virus factories in the cell providing energy for virus release [247]. Similar changes were observed in the chick embryo fibroblasts infected with frog virus 3, where degenerate mitochondria surrounding virus factories were found [248]. 

## 9. Viruses Mimic the Host Mitochondrial Proteins 

 Molecular mimicry is “the theoretical possibility that sequence similarities between foreign and self-peptides are sufficient to result in the cross-activation of autoreactive T or B cells by pathogen-derived peptides” [249, 250]. Since structure follows the function, viruses, during their coevolution with hosts have evolved to mimic the host proteins to meet their ends during progression of their life cycle inside the cell. Mimicking aids the viruses to gain access to host cellular machinery and greatly helps in their survival in the hostile host environment. 

Mimivirus, a member of the newly created virus family *Mimiviridae*, encodes a eukaryotic mitochondria carrier protein (VMC-I) [251], which mimics the host cell's mitochondrial carrier protein and thus controls the mitochondrial transport machinery in infected cells. It helps to transport ADP, dADP, TTP, dTTP, and UTP in exchange for dATP, thus exploiting the host for energy requirements during replication of its A+T rich genome [251]. Besides VMC-I, mimivirus encodes several other proteins (L359, L572, R776, R596, R740, R824 L81, R151, R900, and L908) with putative mitochondria localization signals, which suggest that mimivirus has evolved a strategy to take over the host mitochondria and exploited its physiology to compensate for its energy requirements and biogenesis [251]. Viral Bcl-2 homologues (vBcl-2) are other groups of viral proteins that mimic the host cell Bcl-2s and have been described elsewhere in this review. 

## 10. Viruses Cause Host Mitochondrial DNA Depletion 

 Mammalian mitochondria contain a small circular genome, which synthesizes enzymes for oxidative phosphorylation and mitochondrial RNAs (mtRNAs) [27]. To increase the chance of survival, some viruses appear to have adopted the strategy of damaging the host cell mitochondrial DNA. Since mitochondria act as a source of energy and play an important role in antiviral immunity as well, it is possible that damage to mitochondrial DNA may help in evading mitochondrial antiviral immune responses [252].

During productive infection of mammalian cells *in vitro*, HSV-1 induces the rapid and complete degradation of host mitochondrial DNA [252]. The UL12.5 protein of HSV-1 localizes to the mitochondria and induces DNA depletion in the absence of other viral gene products [252, 253]. The immediate early Zta protein of EBV interacts with mitochondrial single stranded DNA binding protein resulting in reduced mitochondrial DNA (mtDNA) replication and enhanced viral DNA replication [254]. HCV causes the reactive oxygen species and nitrous oxide mediated DNA damage in host mtDNA [107, 255]. Interestingly, depletion of mtDNA has also been observed in HIV/HCV coinfected humans [256].

## 11. Conclusions

Though progress has been made in understanding the interaction of viruses with mitochondria-mediated pathways, the pathways linking the detection of viral infection by PRRs (or exact mechanism by which PRRs recognize the PAMPs) and their link to mitochondria-mediated cell death remain poorly understood. Role of the mitochondria in immunity and viral mechanisms to evade them highlights the fact that even after billions of years of coevolution, the fight for the survival is still going on. Both the host and the viruses are evolving, finding new ways to survive. It may be interesting to note that mitochondria mediated apoptosis might be an evolutionary adaptation by which they might have effectively prevented the entry of other microorganisms trying to gain entry into the host cell and thus effectively establishing themselves as an integral part of the cell.
